# Mrs. Lyman’s Letter

**Published:** 1875-03

**Authors:** Laura E. Lyman


*My dear Sir:* Your letter of inquiry with respect to the healthfulness of Richmond Hill I hasten to answer.

I agree with you entirely that whatever the charms of the locality may be, and however desirable it is as a place of residence on account of its nearness to New York, its neatness, and its picturesque landscape views, unless all sanitary conditions are as they should be, every other attraction is of little consequence.

From photographs and descriptions, no less than from going over the ground in person, you must have a sufficiently clear idea of the outward charms of the place: so I shall give you only what neither the pictures nor your own eyes could assure you of—proof drawn from experience as to the perfect healthfulness of the locality. We have lived here, winter and summer, more than three years, so that I think I can speak with intelligence on this point.

The matter of healthfulness was the first thing to be settled when our coming here was spoken of; for, with a family cf six young children, we felt as you do, that that was of the most vital importance. Mr. Lyman examined carefully the questions of drainage, climatic changes, local causes of disease, etc., and satisfied himself perfectly that, as a country residence, in these, not less than in other respects, Richmond Hill is incomparably more desirable than any other place within thrice its distance from New York; and that, with ordinary obedience to the laws of hygiene, we and our children could be as healthful here as we could be anywhere.

Experience has fully proven the correctness of his judgment. The illnesses we have suffered from have been such as might have overtaken us anywhere; and, on the whole, we have had fewer interruptions of perfect health than for any previous three years in the family history.

Though there have been several deaths in the neighborhood since our residence here, there has but one resulted from disease contracted here; that was a very young child that died of cholera infantum. We have the purest air, the best of water, both cistern and well water, and perfect drainage. A layer of gravel extends a few feet below the surface-soil, from the crest of the hills to the ocean; and should there be water standing anywhere, it is but an hour's work to sink a dry well that shall drain it all away. There remains no improvement of that sort to be made. Every possible sanitary condition has been complied with, and the almost complete immunity from sickness enjoyed by all our residents, children especially, testifies to the perfect healthfulness of the place.

As a home for children it is unsurpassed: the wide range they have in their daily rambles through wood and field, and the absence of all sights and sounds that can affect injuriously, either soul or body, make this a most desirable place for children and young persons; and this, I am sure, will be the universal testimony of all parents here.

Very respectfully,

LAURA E. LYMAN.

---

It is ordained in the eternal constitution of things that men of intemperate minds can not be free; their passions forge their fetters.

